# A normalization strategy for comparing tag count data

**DOI:** 10.1186/1748-7188-7-5

**Published:** 2012-04-05

**Authors:** Koji Kadota, Tomoaki Nishiyama, Kentaro Shimizu

**Affiliations:** 1 Agricultural Bioinformatics Research Unit, Graduate School of Agricultural and Life Sciences, The University of Tokyo, 1-1-1 Yayoi, Bunkyo-ku, Tokyo 113-8657, Japan; 2 Project on Health and Anti-aging, Kanagawa Academy of Science and Technology, 3-2-1 Sakado, Takatsu-ku, Kawasaki, Kanagawa 213-0012, Japan; 3 Advanced Science Research Center, Kanazawa University, 13-1 Takara-machi, Kanazawa 920-0934, Japan

## Abstract

**Background:**

High-throughput sequencing, such as ribonucleic acid sequencing (RNA-seq) and chromatin immunoprecipitation sequencing (ChIP-seq) analyses, enables various features of organisms to be compared through tag counts. Recent studies have demonstrated that the normalization step for RNA-seq data is critical for a more accurate subsequent analysis of differential gene expression. Development of a more robust normalization method is desirable for identifying the true difference in tag count data.

**Results:**

We describe a strategy for normalizing tag count data, focusing on RNA-seq. The key concept is to remove data assigned as potential differentially expressed genes (DEGs) before calculating the normalization factor. Several R packages for identifying DEGs are currently available, and each package uses its own normalization method and gene ranking algorithm. We compared a total of eight package combinations: four R packages (*edgeR*, *DESeq*, *baySeq*, and *NBPSeq*) with their default normalization settings and with our normalization strategy. Many synthetic datasets under various scenarios were evaluated on the basis of the area under the curve (AUC) as a measure for both sensitivity and specificity. We found that packages using our strategy in the data normalization step overall performed well. This result was also observed for a real experimental dataset.

**Conclusion:**

Our results showed that the elimination of potential DEGs is essential for more accurate normalization of RNA-seq data. The concept of this normalization strategy can widely be applied to other types of tag count data and to microarray data.

## Background

Development of next-generation sequencing technologies has enabled biological features such as gene expression and histone modification to be quantified as tag count data by ribonucleic acid sequencing (RNA-seq) and chromatin immunoprecipitation sequencing (ChIP-seq) analyses [1,2]. Different from hybridization-based microarray technologies [3,4], sequencing-based technologies do not require prior information about the genome or transcriptome sequences of the samples of interest [5]. Therefore, researchers can profile the expression of not only well-annotated model organisms but also poorly annotated non-model organisms. RNA-seq in such organisms enables the gene structures and expression levels to be determined.

One important task for RNA-seq is to identify differential expression (DE) for genes or transcripts. Similar to microarray analysis, we typically start the analysis with a so-called "gene expression matrix," where each row indicates the gene (or transcript), each column indicates the sample (or library), and each cell indicates the number of reads mapped to the gene in the sample. In general, there are two major factors for accurately quantifying and normalizing RNA-seq data: gene length and sequencing depth (or total read counts). Normalization by gene length is important for comparing different genes within a sample because longer genes tend to have more reads to be sequenced [6]. Previous approaches for normalizing length include defining an effective length of a gene that may have two or more transcript isoforms of different lengths, and normalizing by the length [7-11].

Normalization by sequencing depth is particularly important for comparing genes in different samples because different samples generally have different total read counts. Previous approaches include (i) global scaling so that a summary statistic such as the mean or upper-quartile value of read counts for each sample (or library) becomes a common value and (ii) standardization of distribution so that the read count distributions become the same across samples [12-15]. Some groups recently reported that over-representation of genes with higher expression in one of the samples, i.e., biased differential expression, has a negative impact on data normalization and consequently can lead to biased estimates of true differentially expressed genes (DEGs) [15,16]. To reduce the effect of such genes on the data normalization step, Robinson and Oshlack reported a simple but effective global scaling method, the trimmed mean of M values (TMM) method, where a scaling factor for the normalization is calculated as a weighted trimmed mean of the log ratios between two classes of samples (i.e., Samples A vs. B) [16]. The concept of the TMM method is the basis for developing our normalization strategy.

In this paper, we focus on normalization related to sequencing depth as well as the TMM normalization method. We believe the TMM method can be improved. Consider, for example, a hypothetical dataset containing a total of 1000 genes, where (i) 200 genes (i.e., 200/1000 = 20%) are detected as DEGs when comparing Samples A vs. B (*P*_DEG _= 20%), (ii) 180 of the 200 DEGs are highly expressed in Sample A (i.e., *P*_A _= 180/200 = 90%), (iii) the dataset can be perfectly normalized by applying a normalization factor calculated based only on the remaining 800 non-DEGs, and (iv) individual DEGs (or non-DEGs) have a negative (or positive) impact on calculation of the normalization factor. In this case, the two parameters should ideally be estimated as *P*_DEG _= 20% and *P*_A _= 90%. Currently, the TMM method implicitly uses fixed values for these two parameters (i.e., *P*_DEG _= 60 and *P*_A _= 50) unless users explicitly provide arbitrary values [16,17]. This is probably because an automatic estimation of the *P*_DEG _value is practically difficult.

Hardcastle and Kelly [18] recently proposed an R [19] package, *baySeq*, for differential expression analysis of RNA-seq data. A notable advantage of this method is that an objective *P*_DEG _value is produced by calculating multiple models of differential expression. This method also inspired us in our improvement of the normalization of RNA-seq data. Our normalization strategy, named TbT, consists of TMM [16] and *baySeq *[18], used twice and once respectively in a TMM-*baySeq*-TMM pipeline. We show the importance of estimating the *P*_DEG _value according to the *true P*_DEG _value for individual datasets. The results were obtained using simulated and real datasets.

## Results and Discussion

RNA-seq data must be normalized before differential expression analysis can be conducted on them. Some R packages exist for comparing two groups of samples [17,18,20,21], and each package uses its own normalization method and gene ranking algorithm. For example, the R package *edgeR *[17] uses the TMM method [16] for data normalization and an exact test for negative binomial (NB) distribution [22] for gene ranking. A good normalization method coupled with gene ranking methods should produce good ranked gene lists where *true *DEGs can easily be detected as top-ranked and non-DEGs are bottom-ranked, when all genes are ranked according to the degree of DE.

Following from our previous study [23-25], the area under the receiver operating characteristic (ROC) curve (i.e., AUC) values were used for evaluating individual combinations based on sensitivity and specificity simultaneously. A good combination should therefore have a high AUC value (i.e., high sensitivity and specificity). In the remainder of this paper, we first describe our normalization strategy (called TbT). We then evaluate a total of eight package combinations: four R packages for differential expression analysis (*edgeR *[17], *DESeq *[20], *baySeq *[18], and *NBPSeq *[21]) with default normalization settings (which we call *edgeR*/default, *DESeq*/default, *baySeq*/default, and *NBPSeq*/default) and the same four packages with TbT normalization (i.e., *edgeR *coupled with TbT (*edgeR*/TbT), *DESeq*/TbT, *baySeq*/TbT, and *NBPSeq*/TbT). Finally, we discuss guidelines for meaningful differential expression analysis.

Note that the execution of the *baySeq *package was performed using data after scaling for the reads per million (RPM) mapped reads in each sample. The procedure in the *baySeq *package and in the other three packages (*edgeR*, *DESeq*, and *NBPSeq*) is not intended for use with RPM-normalized data, i.e., the original raw count data should be used as the input. However, we found that the use of RPM-normalized data generally yields higher AUC values compared to the use of raw count data when executing the *baySeq *package. We also found that the use of RPM data did not positively affect the results when the other three packages were executed. Accordingly, all of the results relating to the *baySeq *package were obtained using the RPM-normalized data. This includes step 2 in the TbT normalization and the gene ranking of DEGs using two *baySeq*-related combinations (*baySeq*/TbT and *baySeq*/default).

### Outline of TbT normalization strategy

The key feature of TbT is that data assigned as potential DEGs are removed before the normalization factor is calculated. We will explain the concept of TbT by using simulation data that are negative binomially distributed (three libraries from Sample A vs. three libraries from Sample B; i.e., {A_1_, A_2_, A_3_} vs. {B_1_, B_2_, B_3_}). The simulation conditions were that (i) 20% of genes were DEGs (*P*_DEG _= 20%), (ii) 90% of *P*_DEG _was higher in Sample A (*P*_A _= 90%), and (iii) the level of DE was four-fold.

The NB model is generally applicable when the tag count data are based on biological replicates. It has been noted that the variance of biological replicate read counts for a gene (*V*) is higher than the mean (*μ*) of the read counts (e.g., *V *= *μ + ϕμ*^2 ^where *ϕ *> 0) and that the extra dispersion parameter *ϕ *tends to have large (or small) values when *μ *is small (or large) [20,21]. To mimic this mean-dispersion relationship in the simulation, we used an empirical distribution of these values (*μ *and *ϕ*) calculated from Arabidopsis data available in the *NBPSeq *package [21]. For details, see the Methods section.

**Figure 1:**
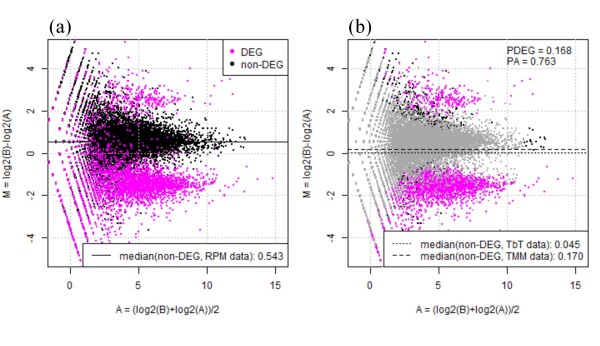
**Outline of TbT normalization strategy**. Left panel: M-A plot for negative binomially distributed simulation data from Ref. [21], after scaling for RPM mapped reads in each sample. Magenta and black dots indicate DEGs (20% of all genes; *P*_DEG _= 20%) and non-DEGs (80%), respectively. 90% of all DEGs is four-fold higher in Sample A than B (*P*_A _= 90%). Each dot represents a gene. Right panel: same plot but colored differently. TbT estimates 16.8% of *P*_DEG _using this data. Gray dots indicate genes estimated as non-DEGs by step 2 in TbT. Note that the median log-ratio for true non-DEGs when data normalization is performed using the TbT normalization factors (0.045) is closer to zero than that using the TMM normalization factors (0.170).

An M-A plot of the simulation data, after scaling for RPM reads in each library, is shown in Figure 1a. The horizontal axis indicates the average expression level of a gene across two groups, and the vertical axis indicates log-ratios (Sample B relative to Sample A). As shown by the black horizontal line, the median log-ratio for non-DEGs based on the RPM-normalized data (0.543) has a clear offset from zero due to the introduced DEGs with the above three conditions. Therefore, the primary aim of our method is to accurately estimate the percentage of true DEGs (*P*_DEG_) and trim the corresponding DEGs so that the median log-ratio for non-DEGs is as close to zero as possible when our TbT normalization factors are used.

To accomplish this, our normalization method consists of three steps: (1) temporary normalization, (2) identification of DEGs, and (3) final normalization of data after eliminating those DEGs. We used the TMM method [16] at steps 1 and 3 and an empirical Bayesian method implemented in the *baySeq *package [18] at step 2. Other methods could have been used, but our choices seemed to produce good ranked gene lists with high sensitivity and specificity (i.e., a high AUC value). We observed that the median log-ratio for non-DEGs based on our TbT normalization factors (0.045) was closer to zero than the log-ratio based on the TMM normalization factors (0.170) that corresponds to the result of TbT right after step 1 (Figure 1b).

This result suggests the validity of our strategy of removing potential DEGs before calculating the normalization factor. Recall that the true values for *P*_DEG _and *P*_A _in this simulation were 20% and 90%, respectively. Our TbT method estimated 16.8% of *P*_DEG _and 76.3% of *P*_A_. We found that 64.4% of the estimated DEGs were true DEGs (i.e., sensitivity = 64.4%) and that the overall accuracy was 89.0%. Some researchers might think that the TMM method (i.e., *P*_DEG _= 60% and *P*_A _= 50%) must be able to remove many more true DEGs than our TbT method (i.e., higher sensitivity). This is true, but the TMM method tends to trim many more non-DEGs than our method (i.e., lower specificity), especially when most DEGs are highly expressed in one of the samples (corresponding to our simulation conditions with high *P*_DEG _and *P*_A _values). These characteristics for the two normalization methods and the results shown in Figure 1 indicate that the balance of sensitivity and specificity regarding the assignment of both DEGs and non-DEGs is critical. Our TbT method was originally designed to normalize tag count data for various scenarios including such biased differential expression.

The successful removal of DEGs in the data normalization step generally increases both the sensitivity and specificity of the subsequent differential expression analysis. Indeed, when an exact test implemented in the R package *edgeR *[17] was used in common for gene ranking, the TbT normalization method showed a higher AUC value (i.e., *edgeR*/TbT = 90.0%) than the default (the TMM method [16] in this package) normalization method (i.e., *edgeR*/default = 88.9%). We also observed the same trend for the other combinations: *DESeq*/TbT = 88.7%, *DESeq*/default = 87.4%, *baySeq*/TbT = 90.2%, *baySeq*/default = 78.2%, *NBPSeq*/TbT = 90.1%, and *NBPSeq*/default = 80.9%. These results also suggest that our TbT normalization strategy can successfully be combined with the four existing R packages and that the TbT method outperforms the other normalization methods implemented in these packages.

### Simulation results

**Figure 2:**
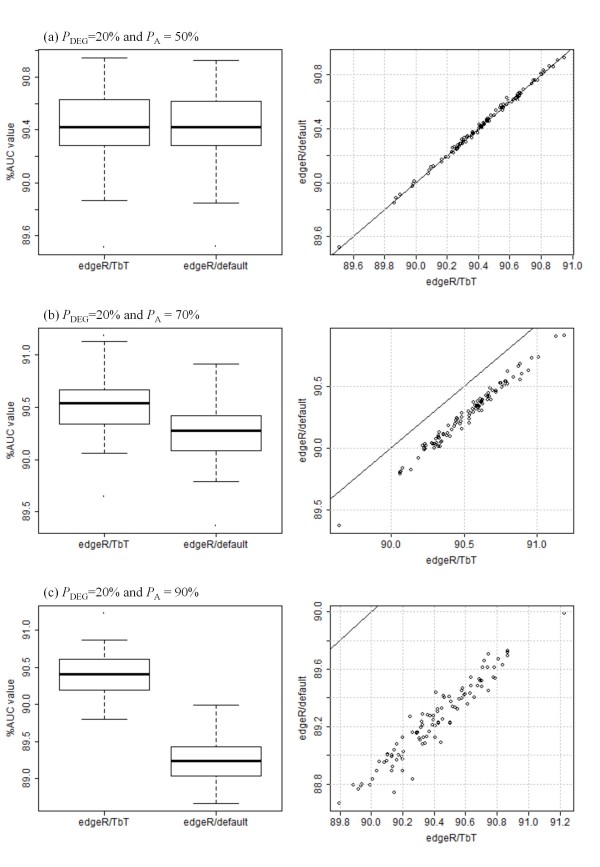
**Distributions of AUC values for two *edgeR*-related combinations**. Simulation results for 100 trials under *P*_A _= (a) 50%, (b) 70%, and (c) 90%, with *P*_DEG _= 20%. Left panel: box plots for AUC values. Right panel: scatter plots for AUC values. When the performances between the two combinations are completely the same, all the points should be on the black (*y *= *x*) line. Point below (or above) the black line indicates that the AUC value from the *edgeR*/TbT combination is higher (or lower) than that from the *edgeR*/default combination.

Note that different trials of simulation analysis generally yield different AUC values even if the same simulation conditions are introduced. It is important to show the statistical significance, if any, of our proposed method. The distributions of AUC values for two *edgeR*-related combinations (*edgeR*/TbT and *edgeR*/default) under three conditions (*P*_A _= 50, 70, and 90% with a fixed *P*_DEG _value of 20%) are shown in Figure 2. The performances between the two combinations were very similar when *P*_A _= 50% (Figure 2a; *p*-value = 0.95, Wilcoxon rank sum test). This is reasonable because the average estimate of the *P*_A _values by TbT in the 100 trials (49.62%) was quite close to the truth (i.e., 50%) and TMM uses a fixed *P*_A _value of 50%. The higher the *P*_A _value (> 50%) TbT estimates, the higher the performance of TbT (compared to TMM) that can be expected.

Different from the above unbiased case (*P*_A _= 50%), we observed the obvious superiority of TbT under the other two conditions (*P*_A _= 70 and 90%). A significant improvement resulting from use of TbT may seem doubtful because of the very small difference between the two average AUC values (e.g., 90.52% for *edgeR*/TbT and 90.26% for *edgeR*/default when *P*_A _= 70%; left panel of Figure 2b), but the *edgeR*/TbT combination did outperform the *edgeR*/default combination in all of the 100 trials under the two conditions (right panels of Figures 2b and 2c), and the *p*-values were lower than 0.01 (Wilcoxon rank sum test).

Table 1 shows the average AUC values for the two *edgeR*-related combinations under the various simulation conditions (*P*_DEG _= 5-30% and *P*_A _= 50-100%). Overall, *edgeR*/TbT performed better than *edgeR*/default for most of the simulation conditions analyzed. The relative performance of TbT compared to the default method (i.e., the TMM method [16] in this case) can be seen to improve according to the increased *P*_A _values starting from 50%. This is because our estimated values for *P*_DEG _and *P*_A _are closer to the *true *values than the fixed values of TMM (*P*_DEG _= 60% and *P*_A _= 50%; see Table 2). The closeness of those estimations will inevitably increase the overall accuracy of assignment for DE and lead directly to the higher AUC values. This success primarily comes from our three-step normalization strategy, TbT (the TMM-*baySeq*-TMM pipeline).

**Table 1:** Average AUC values for two *edgeR*-related combinations.

	*P*_A _= 50%	60%	70%	80%	90%	100%
	(a) *edgeR*/TbT					
*P*_DEG _= 5%	90.52	**89.92**	**90.58**	**90.67**	**90.59**	**90.10**
10%	**90.33**	**90.23**	**90.80**	90.14*	91.02*	90.39*
20%	**90.43**	**90.53**	90.52*	90.60*	90.41*	90.41*
30%	90.71	90.66*	90.23*	90.67*	90.00*	89.46*
	(b) *edgeR*/default					
5%	**90.52**	89.92	90.56	90.62	90.50	89.95
10%	90.33	90.21	90.73	89.99	90.74	89.89
20%	90.43	90.49	90.26	90.00	89.24	88.40
30%	**90.71**	90.54	89.58	89.35	87.20	84.55

Average AUC values of total of 100 trials for each simulation condition: (a) *edgeR*/TbT and (b) *edgeR*/default. Simulation data contain a total of 20,000 genes: *P*_DEG _% of genes is DEGs, and *P*_A _% of *P*_DEG _is higher in Sample A. A total of 24 conditions (four *P*_DEG _values × six *P*_A _values) are shown. Highest AUC value for each condition is in bold. AUC values with asterisks indicate significant improvements (*p*-value < 0.01, Wilcoxon rank sum test).

**Table 2:** Estimated values for *P*_DEG _and *P*_A _by TbT.

	True *P*_A _= 50%	60%	70%	80%	90%	100%
	(a) Estimated *P*_DEG _(%)					
*P*_DEG _= 5%	5.65	5.44	5.68	5.61	5.67	5.54
10%	9.38	9.39	9.58	9.28	9.54	9.31
20%	17.14	17.41	17.21	17.22	17.11	17.01
30%	25.47	25.19	24.87	25.15	24.61	24.34
	(b) Estimated *P*_A _(%)					
5%	49.44	55.08	59.55	65.56	70.02	74.35
10%	50.66	56.27	61.64	67.47	73.98	79.51
20%	49.62	57.41	63.67	69.17	75.49	82.30
30%	50.05	56.58	63.34	70.08	72.47	76.05
	(c) Sensitivity					
5%	62.17	59.53	62.13	62.06	61.79	60.32
10%	63.61	63.41	64.85	62.59	64.27	62.31
20%	67.37	68.15	67.24	66.68	65.13	63.98
30%	71.07	70.09	68.53	68.69	63.99	59.56
	(d) Specificity					
5%	97.35	97.43	97.31	97.39	97.31	97.37
10%	96.70	96.67	96.62	96.69	96.60	96.64
20%	95.53	95.39	95.40	95.26	95.01	94.84
30%	94.25	94.22	94.00	93.67	92.42	90.89
	(e) Accuracy					
5%	95.58	95.52	95.54	95.61	95.52	95.50
10%	93.36	93.31	93.42	93.26	93.34	93.17
20%	89.86	89.90	89.73	89.50	88.99	88.62
30%	87.25	86.94	86.32	86.13	83.84	81.43

Average estimates of total of 100 trials for (a) *P*_DEG _and (b) *P*_A_. The (c) sensitivity, (d) specificity, and (e) accuracy for the estimation are also shown.

Table 3 shows the simulation results for the other six combinations. As can be seen, TbT performed better than the individual default normalization methods implemented in the other three packages (*DESeq *[20], *baySeq *[18], and *NBPSeq *[21]). When we compare the results of the four default procedures (*edgeR*/default, *DESeq*/default, *baySeq*/default, and *NBPSeq*/default), the *edgeR*/default combination outperforms the others. This result suggests the superiority of the default normalization method (i.e., TMM) implemented in the *edgeR *package and the validity of our choices at steps 1 and 3 in our TbT normalization strategy. For reproducing the research, the R-code for obtaining a small portion of the above results is given in Additional file 1.

**Table 3:** Average AUC values for other six combinations.

	*P*_A _= 50%	60%	70%	80%	90%	100%
	(a) *DESeq*/TbT					
*P*_DEG _= 5%	**85.03**	**83.94**	**85.20**	**85.31**	85.12*	84.60*
10%	**86.94**	**86.90**	87.42*	86.80*	87.61*	86.95*
20%	89.05	**89.23**	89.18*	89.33*	88.97*	88.95*
30%	**90.30**	90.20*	89.79*	90.11*	89.44*	88.80*

	(b) *DESeq*/default					
5%	85.03	83.92	85.13	85.19	84.84	84.18
10%	86.93	86.85	87.27	86.46	87.07	86.15
20%	**89.06**	89.19	88.93	88.62	87.76	86.84
30%	90.30	90.00	88.94	87.95	85.36	81.98

	(c) *baySeq*/TbT					
5%	**89.91**	**89.45**	89.91*	90.17*	89.93*	89.36*
10%	**89.89**	89.90*	90.46*	89.79*	90.28*	90.02*
20%	90.39*	90.46*	90.40*	90.49*	90.21*	90.47*
30%	**90.80**	90.55*	90.44*	90.69*	89.26*	88.33*

	(d) *baySeq*/default					
5%	89.67	89.27	88.62	88.69	86.37	86.18
10%	89.80	89.55	89.52	87.71	84.14	83.86
20%	90.22	88.78	88.92	87.85	79.09	69.65
30%	90.76	90.05	87.21	79.69	65.45	53.37

	(e) *NBPSeq*/TbT					
5%	90.75*	**90.18**	90.80*	90.90*	90.78*	90.33*
10%	90.59*	90.47*	91.00*	90.34*	91.14*	90.49*
20%	90.67*	90.72*	90.70*	90.68*	90.42*	90.37*
30%	**90.92**	90.83*	90.32*	90.74*	89.89*	89.23*

	(f) *NBPSeq*/default					
5%	90.48	90.00	89.71	89.58	87.85	87.60
10%	90.34	90.15	90.11	88.46	86.19	85.38
20%	90.39	89.12	89.22	88.29	81.59	73.93
30%	90.84	90.26	87.45	81.96	70.97	60.73

Results for (a) *DESeq*/TbT, (b) *DESeq*/default, (c) *baySeq*/TbT, (d) *baySeq*/default, (e) *NBPSeq*/TbT, and (f) *NBPSeq*/default. Higher AUC values between different normalization methods in each package are in bold. AUC values with asterisks indicate significant improvements (*p*-value < 0.01, Wilcoxon rank sum test).

Recall that the level of DE for DEGs was four-fold in this simulation framework and the shape of the distribution for introduced DEGs is the same as that of non-DEGs (left panel of Figure 1). This indicates that some DEGs introduced as higher expression in Sample A (or Sample B) can display positive (or negative) M values even after adjustment by the median M value for non-DEGs. In other words, there are some DEGs whose log-ratio signs (i.e., directions of DE) are different from the original intentions. Although the simulation framework regarding the introduction of DEGs was the same as that described in the TMM study [16], this may weaken the validity of the current simulation framework.

To mitigate this concern, we performed simulations with compatible directions of DE by adding a floor value of fold-changes (> 1.2-fold) when introducing DEGs. In this simulation, the fold-changes for DEGs were randomly sampled from "1.2 + a gamma distribution with shape = 2.0 and scale = 0.5." Accordingly, the minimum and mean fold-changes were approximately 1.2 and 2.2 (= 1.2 + 2.0 × 0.5), respectively. We confirmed the superiority of TbT under the various simulation conditions (*P*_DEG _= 5-30% and *P*_A _= 50-100%) with the above simulation framework (data not shown). An M-A plot of the simulation result when *P*_DEG _= 20% and *P*_A _= 90% is given in Additional file 2. The R-code for obtaining the full results under the simulation condition is given in Additional file 3.

### Iterative normalization approach

**Figure 3:**
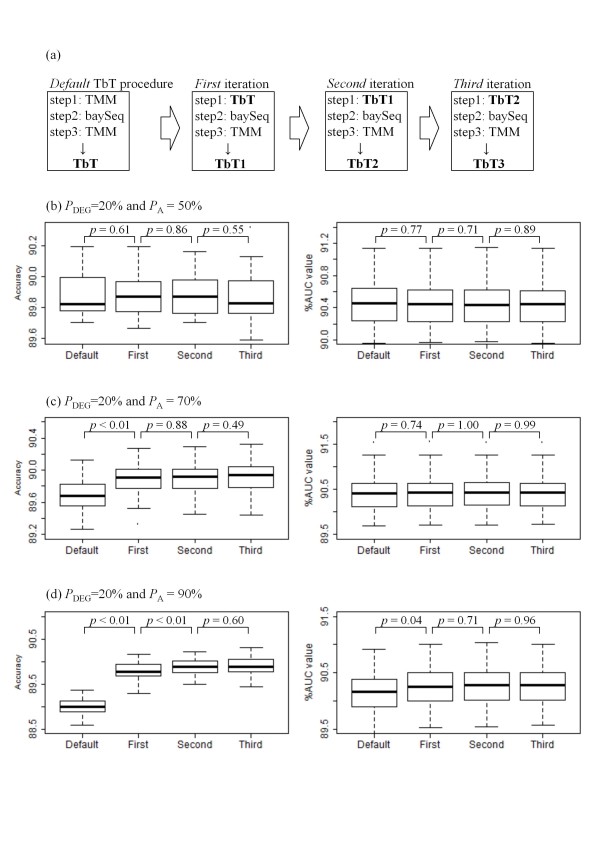
**Results of iterative TbT approach**. (a) Procedure for iterative TbT approach until the third iteration, and simulation results under *P*_A _= (b) 50%, (c) 70%, and (d) 90%, with *P*_DEG _= 20%. Left panel: accuracies of DEG identifications when step 2 in our DEG elimination strategy is performed using the following normalization factors: TMM (*Default*), TbT (*First*), TbT1 (*Second*), and TbT2 (*Third*). Right panel: AUC values when the following normalization factors are combined with the *edgeR *package: TbT (*Default*), TbT1 (*First*), TbT2 (*Second*), and TbT3 (*Third*).

Recall that the outperformance of TbT compared to TMM (see Table 1 and Figure 2) is by virtue of our DEG elimination strategy for normalizing tag count data and that the identification of DEGs in TbT is performed using *baySeq *with the TMM normalization factors at step 2. From these facts, it is expected that the accuracy of the DEG identification at step 2 can be increased by using *baySeq *with the TbT factors instead of the TMM factors when *P*_A _> 50%. The advanced DEG elimination procedure (the TbT-*baySeq*-TMM pipeline) can produce different normalization factors (say "TbT1") from the original ones. As also illustrated in Figure 3a, this procedure can repeatedly be performed until the calculated normalization factors become convergent.

The results under three simulation conditions (*P*_A _= 50, 70, and 90% with a fixed *P*_DEG _value of 20%) are shown in Figures 3b-d. The left panels show the accuracies of DEG identifications when step 2 in our DEG elimination procedures is performed using the following normalization factors: TMM (*Default*), TbT (*First*), TbT1 (*Second*), and TbT2 (*Third*). As expected, the iterative approach does not positively affect the results when *P*_A _= 50% (Figure 3b). Indeed, the performances between the *baySeq*/TMM combination (*Default*) and the *baySeq*/TbT2 combination (*Third*) are not statistically distinguished (*p *= 0.38, Wilcoxon rank sum test). Meanwhile, the use of the *baySeq*/TbT combination (*First*) can clearly increase the accuracy compared to use of the *baySeq*/TMM combination (*Default*), though the subsequent iterations do not improve the accuracies when *P*_A _= 70% (Figure 3c, left panel). An advantageous trend for the iterative approach was also observed until the second iteration (*Second*; the *baySeq*/TbT1 combination) when *P*_A _= 90% (Figure 3d, left panel).

The right panels for Figures 3b-d show the AUC values when the following normalization factors are combined with the *edgeR *package: TbT (*Default*), TbT1 (*First*), TbT2 (*Second*), and TbT3 (*Third*). The overall trend is the same as that of the accuracies shown in the left panels: the iterative TbT approach can outperform the original TbT approach when the degree of biased differential expression is high (*P*_A _> 50%). We confirmed the utility of the iterative approach with the other three packages (*DESeq*, *baySeq*, and *NBPSeq*) (data not shown). These results suggest that the iterative approach can be recommended, especially when the *P*_A _value estimated by the original TbT method is displaced from 50%.

Nevertheless, we should emphasize that the improvement of the iterative TbT approach compared to the original TbT approach is much smaller than that of the TbT compared to the default normalization methods implemented in the four R packages investigated (Figures 2 and 3). For example, the average difference of the AUC values between the *edgeR*/TbT3 and the *edgeR*/TbT is 0.02% (Figure 3c) while the average difference of the AUC values between the *edgeR*/TbT and the *edgeR*/default is 0.26% (Figure 2b), when *P*_A _= 70%. Note also that the *baySeq *package used in step 2 in our TbT method is much more computationally intensive than the other three packages, indicating that the *n *times iteration of TbT roughly requires *n*-fold computation time. In this sense, a speed-up of our proposed DEG elimination strategy should be performed next as future work. The R-code for obtaining a small portion of the above results is given in Additional file 4.

### Real data (wildtype vs. *RDR6 *knockout dataset used in *baySeq *study)

Finally, we show results from an analysis similar to that described in Ref. [18]. In brief, Hardcastle and Kelly compared two wildtype and two *RNA-dependent RNA polymerase 6 *(*RDR6*) knockout *Arabidopsis thaliana *leaf samples by sequencing small RNAs (sRNAs). From a total of 70,619 unique sRNA sequences, they identified 657 differentially expressed (DE) sRNAs that uniquely match tasRNA, which is produced by *RDR6*, and that are decreased in *RDR6 *mutants and regarded as provisional true positives. Therefore, we assume that the logical values for *P*_DEG _and *P*_A _are at least 0.93% (= 657/70,619) and around 100%, respectively. In accordance with that study [18], the evaluation metric here is that a good method should be able to rank those true positives as highly as possible. Recall that the strategy for TbT is to normalize data after the elimination of such DE sRNAs for such a purpose.

The TbT estimated 9.0% of *P*_DEG _(5,495 *potential *DE sRNAs) and 70.2% of *P*_A_. We found that the 5,495 sRNAs included 255 of the 657 true positives. This suggests that our strategy was effective because the original percentage (657/70,619 = 0.93%) of true positives decreased ((657 - 255)/(70,619 - 5,495) = 0.62%) before the TbT normalization factor was calculated at step 3. In summary, the TbT normalization factor was calculated based on 65,124 (= 70,619 - 5,495) potentially non-DE sRNAs after 255 out of the 657 provisional DE sRNAs were eliminated.

**Figure 4:**
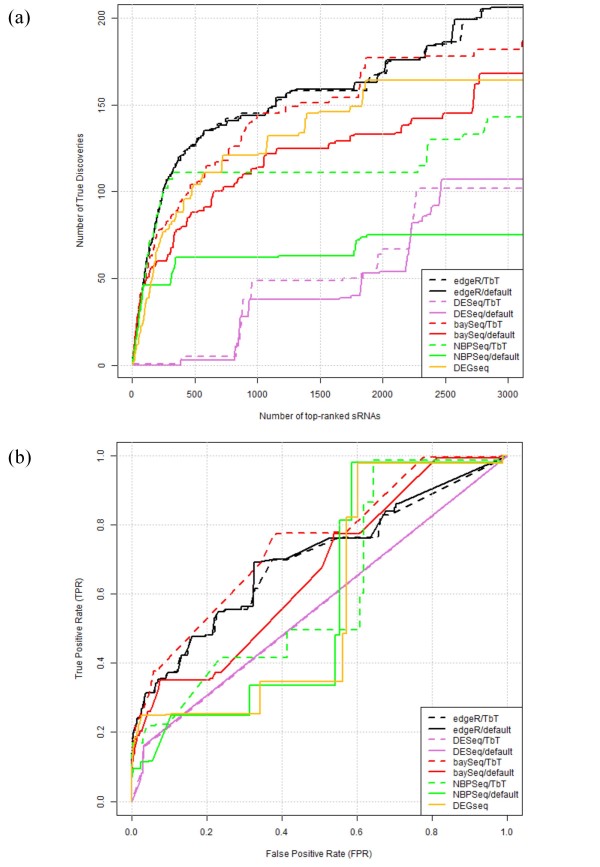
**Results for real data**. (a) Number of tasRNA-associated sRNAs (i.e., provisional true discoveries) for given numbers of top-ranked sRNAs obtained from individual combinations. Combinations of individual R packages with TbT and default normalization methods are indicated by dashed and solid lines, respectively. For easy comparison with the previous study, results of *DEGseq *with the same parameter settings as in the previous study are also shown (solid yellow line). (b) Full ROC plots. Plots on left side (roughly the [0.00, 0.05] region on the *x*-axis) are essentially the same as those shown in Figure 4a. The R-code for producing Figure 4 is available in Additional file 5.

A true discovery plot (the number of provisional true positives when an arbitrary number of top-ranked sRNAs is selected as differentially expressed) is shown in Figure 4a. Note that this figure is essentially the same as Figure five in Ref. [18], so we chose the colors for indicating individual R packages and the ranges for both axes to be as similar as possible to the original. Since the original study [18] reported that another package (*DEGseq *[26]) was the best when the range in the figure was evaluated, we also analyzed the package with the same parameter settings as in Ref. [18] and obtained a reproducible result for *DEGseq*.

Three combinations (*baySeq*/TbT, *edgeR*/TbT, and *edgeR*/default) outperformed the *DEGseq *package. The higher performances of these combinations were also observed from the full ROC curves (Figure 4b). The *baySeq*/TbT combination displayed the highest AUC value (74.6%), followed by *edgeR*/default (70.0%) and *edgeR*/TbT (69.3%). Recall that the *edgeR*/default combination uses the TMM normalization method [16] and that the basic strategy (i.e., potential DEGs are not used) for data normalization is essentially the same as that of our TbT. This result confirms the previous findings [15,16]: potential DE entities have a negative impact on data normalization, and their existences themselves consequently interfere with their opportunity to be top-ranked.

Three combinations (*edgeR*/default, *DESeq*/default, and *baySeq*/default) performed differently between the current study and the original one [18]. The difference for the first two combinations can be explained by the different choices for the *default *normalization methods. Hardcastle and Kelly [18] used a simple normalization method by adjusting the total number of reads in each library for both packages with a reasonable explanation for why the recommended method (i.e., the default method we used here) implemented in the *DESeq *package was not used. The TMM normalization method that we used as the *default *in the *edgeR *package was probably not implemented in the package when they conducted their evaluation. We found that both procedures (i.e., *edgeR *and *DESeq *packages with library-size normalization) performed poorly on average (data not shown).

The difference between the current result (*baySeq*/default; solid red line in Figure 4a) and the previous result (dashed red line in Figure five in Ref. [18]) might be explained by the fact that bootstrap resampling was conducted a different number of times for estimating the empirical distribution on the parameters of the NB distribution. Although the current result was obtained using 10,000 iterations of resampling as suggested in the package, we sometimes obtained a similar result to the previous one when we analyzed *baySeq*/default using 1,000 iterations of resampling. We therefore determined that the previous result was obtained by taking a small sample, such as 1,000 iterations. In any case, we found that those results for the *baySeq*/default combination with different parameter settings were overall inferior to the *baySeq*/TbT combination. For reproducing the research, the R-code for obtaining the results in Figure 4 and AUC values for individual combinations is given in Additional file 5.

## Conclusion

We described a strategy (called TbT as an acronym for the TMM-*baySeq*-TMM procedure) for normalizing tag count data. We evaluated the feasibility of TbT based on three commonly used R packages (*edgeR*, *DESeq*, and *baySeq*) and a recently published package *NBPSeq*, using a variety of simulation data and a real dataset. By comparing the default procedures recommended in the individual packages (*edgeR*/default, *DESeq*/default, *baySeq*/default, and *NBPSeq*/default) and procedures where our proposed TbT was used in the normalization step instead of the default normalization method (*edgeR*/TbT, *DESeq*/TbT, *baySeq*/TbT, and *NBPSeq*/TbT), the effectiveness of TbT has been suggested for increasing the sensitivity and specificity of differential expression analysis of tag count data such as RNA-seq.

Our study demonstrated that the elimination of potential DEGs is essential for obtaining good normalized data. In other words, the elimination of the DEGs before data normalization can increase both sensitivity and specificity for identifying DEGs. Conventional approaches consisting of two steps (i.e., data normalization and gene ranking) cannot accomplish this aim in principle. The two-step approach includes the default procedures recommended in individual packages (*edgeR*/default, *DESeq*/default, *baySeq*/default, and *NBPSeq*/default). Our proposed approach consists of a total of four steps (data normalization, DEG identification, data normalization, and DEG identification). This procedure enables potential DEGs to be eliminated before the second normalization (step 3).

Our TbT normalization strategy is a proposed pipeline for the first three steps, where the TMM normalization method is used at steps 1 and 3 and the empirical Bayesian method implemented in the *baySeq *package is used at step 2. This is because our strategy was originally designed to improve the TMM method, the default method implemented in the *edgeR *package. As demonstrated in the current simulation results comparing two groups (for example, samples A and B), the use of default normalization methods implemented in the existing R packages performed poorly in simulations where almost all the DEGs are highly expressed in Sample A (i.e., the case of *P*_A _> > 50% when the range is defined as 50% ≤ *P*_A _≤ 100%). Although the negative impact derived from such biased differential expression gradually increases according to the increased proportion of DEGs in the data, our strategy can eliminate some of those DEGs before data normalization (Tables 1, 2, and 3). The use of the empirical Bayesian method implemented in the *baySeq *package primarily contributes to solving this problem.

Although we focused on expression-level data in this study, similar analysis of differences in ChIP-seq tag counts would benefit from this method. It is natural to expect that loss of the function of histone modification enzymes will lead to biased distribution of the difference between compared conditions in the corresponding ChIP-seq analysis, in a similar way to the *RDR6 *case. We observed relatively high performances for *NBPSeq*/TbT when analyzing simulation data (Tables 1 and 3) and *baySeq*/TbT when analyzing a real dataset (Figure 4). However, this might simply be because the simulation and real data used in this study were derived from the *NBPSeq *study [21] and the *baySeq *study [18], respectively. In this sense, the *edgeR*/TbT combination might be suitable because it performed comparably to the individual bests. The DEG elimination strategy we proposed here could be applied for many other combinations of methods, e.g., the use of an exact test for NB distribution [22] for detecting potential DEGs at step 2. A more extensive study with other recently proposed methods (e.g., Ref. [27]) based on many real datasets should still be performed.

## Methods

All analyses were basically performed using R (ver. 2.14.1) [19] and Bioconductor [28].

### Simulation details

The negative binomially distributed simulation data used in Tables 1, 2, and 3 and Figures 1, 2, and 3 were produced using an R generic function *rnbinom*. Each dataset consisted of 20,000 genes × 6 samples (3 of Sample A vs. 3 of Sample B). Of the 20,000 genes, the *P*_DEG _% were DEGs at the four-fold level, and *P*_A _% of the *P*_DEG _% was higher in Sample A. For example, the simulation condition for Figure 1 used 20% of *P*_DEG _and 90% of *P*_A_, giving 4,000 (= 20,000 × 0.20) DEGs, 3,600 (= 20,000 × 0.20 × 0.9) of which are highly expressed in Sample A in the simulation dataset.

The variance of the NB distribution can generally be modelled as *V *= *μ *+ *ϕμ*^2^. The empirical distribution of read counts for producing the mean (*μ*) and dispersion (*ϕ*) parameters of the model was obtained from Arabidopsis data (three biological replicates for both the treated and non-treated samples) in Ref. [21]. The simulations were performed using a total of 24 combinations of *P*_DEG _(= 5, 10, 20, and 30%) and *P*_A _(= 50, 60, 70, 80, 90, and 100%) values. The full R-code for obtaining the simulation data is described in Additional file 1. The parameter *param1 *in Additional file 1 corresponds to the degree of fold-change.

Simulations with different types of DEG distribution were also performed in this study. The fold-change values for individual genes were randomly sampled from a gamma distribution with shape and scale parameters. Specifically, an R generic function *rgamma *with respective values of 2.0 and 0.5 for the shape and scale parameters was used. This roughly gives respective values of 0.0 and 1.0 for the minimum and mean fold-changes. We added an offset value of 1.2 to prevent low fold-changes for introduced DEGs, giving respective values of 1.2 and 2.2 for the minimum and mean fold-changes. The full R-code for obtaining the simulation data is described in Additional file 3. The values in *param1 *in Additional file 3 correspond to those parameters.

### Wildtype vs. *RDR6 *knockout dataset used in *baySeq *study

The dataset was obtained by e-mail from the author of Ref. [18]. The dataset (named "rdr6_wt.RData") consists of 70,619 sRNAs × 4 samples (2 wildtype and 2 *RDR6 *knockout samples). Of the 70,619 sRNAs, 657 were used as true DE sRNAs whose expressions were higher in the wildtype than the *RDR6 *knockout samples.

### Gene ranking with default procedure

Ranked gene lists according to the differential expression are pre-required for calculating AUC values. The input data for differential expression analysis using five R packages (*edgeR *ver. 2.4.1, *DESeq *ver. 1.6.1, *baySeq *ver. 1.8.1, *NBPSeq *ver. 0.1.4, and *DEGseq *ver. 1.6.2) is basically the raw count data where each row indicates the gene (or transcript), each column indicates the sample (or library), and each cell indicates the number of reads mapped to the gene in the sample. The execution of the *baySeq *package was performed using data after scaling for RPM mapped reads.

The analysis using the *edgeR *packages with default settings (i.e., the *edgeR*/default combination) was performed using four functions (*calcNormFactors*, *estimateCommonDisp*, *estimateTagwiseDisp*, and *exactTest*) in the package [17]. The TMM normalization factor can be obtained from the output object after applying the *calcNormFactors *function [16]. The genes were ranked in ascending order of the *p*-values.

The *DESeq*/default combination was performed using three functions (*estimateSizeFactors*, *estimateDispersions*, and *nbinomTest*) in the package. The genes were ranked in ascending order of the *p*-values adjusted for multiple-testing with the Benjamini-Hochberg procedure.

The *baySeq*/default combination was performed using two functions (*getPriors.NB *and *getLikelihoods.NB*) in the package [18] for the RPM data. The empirical distribution on parameters of the NB distribution was estimated by bootstrapping from the data. We took sample sizes of (i) 2,000 iterations for the simulation data shown in Tables 1, 2, and 3, Figures 1 and 2, and Additional file 2 (see Additional files 1 and 3), (ii) 5,000 iterations for the simulation data shown in Figure 3 (Additional file 4), and (iii) 10,000 iterations for real data (Additional file 5). The genes were ranked in descending order of the posterior likelihood of the model for differential expression.

The *NBPSeq*/default combination was performed using the *nbp.test *function in the package [21]. The genes were ranked in ascending order of the *p*-values of the exact NB test.

The analysis using the *DEGseq *package [26] was performed for benchmarking the current study and a previous study [18], both of which analyzed the same real dataset. There are multiple methods in the *DEGseq *package [26]. Following from the previous study, we used an MA plot-based method with random sampling (MARS), i.e., the *DEGexp *function with method = "MARS" option was used. A higher absolute value for the statistics indicates a higher degree of differential expression. Accordingly, the genes were ranked in descending order of the absolute value. Note that the execution of this package (ver. 1.6.2) was performed using R 2.13.1 because we encountered an error when executing the more recent version (ver. 1.8.0) using R 2.14.1.

### TbT normalization strategy

Our proposed strategy is an analysis pipeline consisting of three steps. In step 1, the TMM normalization factors are calculated by using the *calcNormFactors *function in the *edgeR *package with the raw count data. These factors are used for calculating *effective *library sizes, i.e., library sizes multiplied by the TMM factors.

In step 2, potential DEGs are identified by using the *baySeq *package with the RPM data. Different from the above *baySeq*/default combination, the analysis is performed using the effective library sizes. The effective library sizes are introduced when constructing a *countData *object, the input data for the *getPriors.NB *function. By applying the subsequent *getLikelihoods.NB *function, the percentage of DEGs in the data (the *P*_DEG _value) and the corresponding potential DEGs can be obtained.

In step 3, TMM normalization factors are again calculated based on the raw count data after eliminating the estimated DEGs. The TbT normalization factors are defined as (the TMM normalization factors calculated in this step) × (library sizes after eliminating the DEGs)/(library sizes before eliminating the DEGs). As the TbT normalization factors are comparable with the original TMM normalization factors such as those calculated in step 1, effective library sizes can also be calculated by multiplying library sizes by the TbT factors.

The four combinations coupled with the TbT normalization strategy (*edgeR*/TbT, *DESeq*/TbT, *baySeq*/TbT, and *NBPSeq*/TbT) were analyzed to compare the above four combinations coupled with the default normalization strategy. The *edgeR*/TbT combination introduced the TbT normalization factors instead of the original TMM factors. The *NBPSeq*/TbT combination introduced the TbT normalization factors in the *nbp.test *function. The remaining two combinations (*DESeq*/TbT and *baySeq*/TbT) introduced the effective library sizes, i.e., the original library sizes multiplied by the TbT factors.

## List of abbreviations used

DE: differential expression; DEG: differentially expressed gene; EB: embryonic body; RPM: reads per million (normalization); sRNA: small RNA; tasRNA: *TAS *locus-derived small RNA; TMM: trimmed mean of M values (method).

## Competing interests

The authors declare that they have no competing interests.

## Authors' contributions

KK performed analyses and drafted the paper. TN provided helpful comments and refined the manuscript. KS supervised the critical discussion. All the authors read and approved the final manuscript.

## Supplementary Material

Additional file 1 **R-code for simulation analysis**. After execution of this R-code with default parameter settings (i.e., *rep_num *= 100, *param1 *= 4,.., and *param6 *= 090), two output files named "Fig1.png" and "resultNB_020_090.txt" can be obtained. The former is the same as Figure 1. The latter output file will contain raw data for Tables 1, 2, 3 when *P*_DEG _= 20% and *P*_A _= 90%. The numbers given as *rep_num*, *param1*,..., and *param6 *indicate the number of trials (*rep_num*), degree of differential expression of fold-change (*param1*-fold), number of libraries for sample A (*param2*), number of libraries for sample B (*param3*), total number of genes (*param4*), true *P*_DEG _(*param5*), and true *P*_A _(*param6*), respectively. Accordingly, for example, respective values for *param5 *and *param6 *should be changed to "030" and "060", to obtain the raw results when *P*_DEG _= 30% and *P*_A _= 60%. Click here for file

Additional file 2 **Result of TbT using simulation data with > 1.2-fold of DEGs**. Legends in this figure are essentially the same as those described in Figure 1. The difference between the two is the distributions of DEGs (magenta dots). This simulation does not have DEGs with low fold-changes (< = 1.2-fold) and the average fold-change is theoretically 2.2. The R code for obtaining the full results under the simulation condition (i.e., *P*_DEG _= 20% and *P*_A _= 90%) is given in Additional file 3. Click here for file

Additional file 3 **R-code for obtaining simulation results with > 1.2-fold of DEGs**. After execution of this R-code with default parameter settings (i.e., *rep_num *= 100, *param1 *= c(1.2, 2.0, 0.5),..., and *param6 *= 090), two output files named "Additional2.png" and "resultNB2_020_090.txt" can be obtained. The former is the same as Additional file 2. The format of the latter output file is essentially the same as the "resultNB_020_090.txt" file obtained by executing Additional file 1. The main difference between the current code and Additional file 1 is in the parameter settings for producing the distributions of DEGs at *param1*. The parameter values (1.2, 2.0, and 0.5) indicated in *param1 *are used for the minimum fold-change (= 1.2) and for random sampling of fold-change values from a gamma distribution with shape (= 2.0) and scale (= 0.5) parameters, respectively. Click here for file

Additional file 4 **R-code for obtaining raw results shown in Figure **3. After execution of this R-code with default parameter settings (i.e., *rep_num *= 100, *param1 *= 4,..., and *param7 *= 5000), four output files named "iteration0_020_090.txt", "iteration1_020_090.txt", "iteration2_020_090.txt", and "iteration3_020_090.txt" can be obtained. The box plots for *Default*, *First*, *Second*, and *Third *shown in Figure 3 are produced using values in two columns (named "accuracy" and "AUC(edgeR/TbT)") in the first, second, third, and fourth file, respectively. The *p*-values were calculated based on the Wilcoxon rank sum test. Click here for file

Additional file 5 **R-code for producing Figure **4**and AUC values for individual combinations**. We obtained an input file (named "rdr6_wt.RData") from Dr. T.J. Hardcastle (the corresponding author of Ref. [18]). After execution of this R-code, three output files (arbitrarily named "Fig4a.png", "Fig4b.png", and "AUCvalue_Fig4b.txt") can be obtained. Click here for file
