# Supplementary figures and images for: A normalization strategy for comparing tag count data
Source: Algorithms Mol Biol. 2012 Apr 5;7:5. doi: 10.1186/1748-7188-7-5 (PMC3341196; doi:10.1186/1748-7188-7-5)

## Slide 1
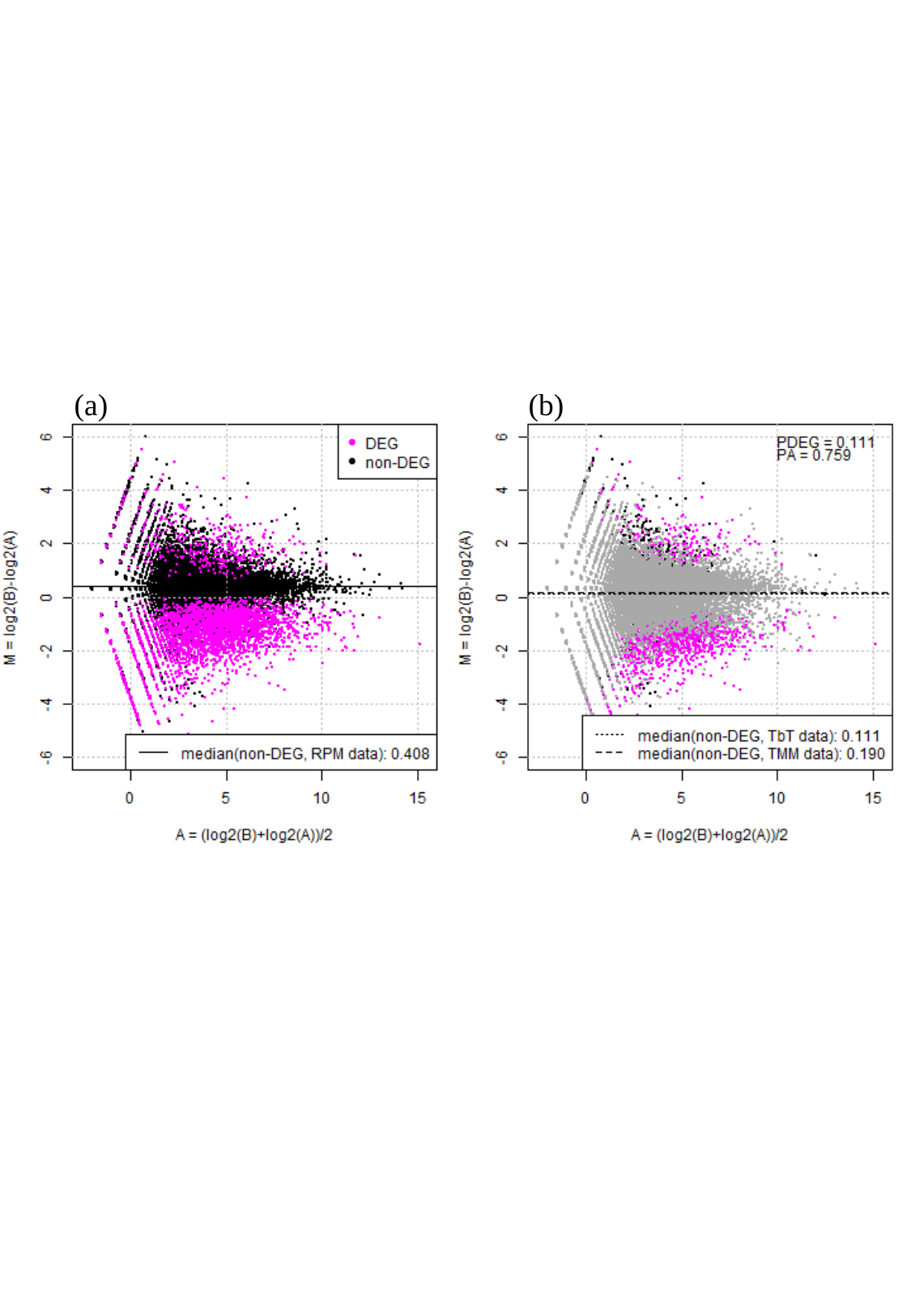

(a)
(b)

Supplement: Additional file 2 — Result of TbT using simulation data with > 1.2-fold of DEGs. Legends in this figure are essentially the same as those described in Figure 1. The difference between the two is the distributions of DEGs (magenta dots). This simulation does not have DEGs with low fold-changes (< = 1.2-fold) and the average fold-change is theoretically 2.2. The R code for obtaining the full results under the simulation condition (i.e., PDEG = 20% and PA = 90%) is given in Additional file 3. [file 1748-7188-7-5-S2.PPT]
